# Interaction with CYP20–3 limits OPDA flux into jasmonate biosynthesis in wounded leaves of arabidopsis grown at eCO_2_

**DOI:** 10.3389/fpls.2026.1725249

**Published:** 2026-04-07

**Authors:** Amelia Redl, Jennifer Gabriel, Nicole M. van Dam, Jacqueline C. Bede

**Affiliations:** 1 Department of Plant Science, McGill University, Sainte-Anne-de-Bellevue, QC, Canada; 2 German Centre for Integrative Biodiversity Research (iDiv) Halle-Jena-Leipzig, Leipzig, Germany; 3 Institute of Biodiversity, Ecology and Evolution (IBEE), Friedrich Schiller University Jena, Jena, Germany; 4 Leibniz Institute of Vegetable and Ornamental Crops (IGZ), Großbeeren, Germany

**Keywords:** 12-*oxo*-phytodienoic acid, CYP20-3, elevated carbon dioxide, jasmonate, salicylic acid, wound stress

## Abstract

Upon recognition of a stress, such as wounding, a rapid increase in jasmonates leads to plant resistance against necrotrophic pathogens and chewing insect herbivores. This jasmonate burst is weaker in *Arabidopsis thaliana* plants grown under future predicted carbon dioxide levels (eCO_2_) compared to todays’ levels. Even though foliar levels of jasmonoyl-isoleucine are lower in wounded arabidopsis at eCO_2_, levels of their precursor, 12-*oxo*-phytodienoic acid (OPDA), are not affected by atmospheric CO_2_ levels. We focused on the role of the OPDA-binding protein CYP20-3 in regulating jasmonate levels in wounded rosettes of arabidopsis grown at eCO_2_. By comparing phytohormone and transcriptomic responses of wounded wildtype plants and *cyp20–3* grown under different CO_2_ conditions, our results suggest that under eCO_2_, CYP20–3 binds to OPDA to limit flux into jasmonate biosynthesis. As well, the CYP20-3-OPDA-SAT1-OASTL-B complex activates cysteine production which can lead to glutathione biosynthesis to buffer changes in the cellular redox state, dampening wound-associated oxidative stress that leads to jasmonate biosynthesis.

## Introduction

Atmospheric carbon dioxide (CO_2_) concentrations are predicted to double, reaching 800–1000 ppm, by 2100, compared to 425 ppm in August 2025 (Lee et al., 2021; https://gml.noaa.gov/ccgg/trends/mlo.html). Even though elevated CO_2_ (eCO_2_) levels are predicted to increase photosynthetic efficiency in C3 plants (Nowak et al., 2004; Leakey et al., 2009), research suggests that C_3_ plants may become more susceptible to necrotrophic pathogens and chewing insect herbivores at eCO_2_ (Bazinet et al., 2022; Bede and Blande, 2025). Levels of jasmonates, such as jasmonic acid (JA) and jasmonoyl-isoleucine (JA-Ile), critical phytohormones in plant resistance against these pathogens and pests (Koo and Howe, 2009; Li et al., 2022), are lower in wounded *Arabidopsis thaliana* plants grown at eCO_2_ (Martinez Henao et al., 2020). However, the mechanism underlying this attenuation is poorly understood.

Wounding, such as that caused by insect chewing herbivory, or recognition of necrotrophic pathogens, triggers jasmonate biosynthesis that leads to induced plant resistance (Wasternack and Hause, 2013; Li et al., 2022). From chloroplast-derived galactolipids, α-linolenic acid leads to the biosynthesis of 12-*oxo*-phytodienoic acid (OPDA) through the action of a 13*S*-lipoxygenase, allene oxide synthase and allene oxide cyclase. OPDA is transported to the peroxisome where it is converted to JA, through β-oxidation and reduction reactions. JA is exported to the cytosol and conjugated to isoleucine to produce jasmonoyl-isoleucine (JA-Ile) which enters the nucleus and binds to the Skp1/Cullin/F-box (SCF)^COI1^ ubiquitin ligase complex and jasmonate-Zim domain (JAZ) proteins (Chini et al., 2009). The bridging of the ubiquitin ligase and JAZ proteins leads to their degradation through the 26S-proteasome and releases MYC2, 4 or 6 transcription factors from repression leading to JA-Ile-responsive gene expression (Wasternack and Strnad, 2018). This results in the expression of many plant resistance-related genes, many of which encode enzymes required for the biosynthesis of specialized metabolites involved in plant defense as well as those involved in jasmonate biosynthesis (Jung et al., 2007). However, it should be noted that in addition to its role as a precursor to JA-Ile, OPDA is also biologically active and regulates the expression of defense genes distinct from those induced by JA-Ile (Taki et al., 2005). However, recent evidence has shown that while treatment with exogenous OPDA activates JA-Ile-independent gene expression, endogenous OPDA produced after wounding functions primarily as a jasmonate precursor rather than as an independent signal (Mekkaoui et al., 2025).

There is growing evidence that jasmonate biosynthesis may be affected by atmospheric CO_2_ levels (Bazinet et al., 2022 and references therein). Of particular note, though lower JA and/or JA-Ile levels are often observed in C3 plants grown at eCO_2_, little effect is seen on the levels of their biosynthetic precursor OPDA. This implies that a CO_2_-mediated block may occur between OPDA and JA biosynthesis.

OPDA is also involved in regulating redox signaling through its interaction with Cyclophilin 20-3 (CYP20-3), an OPDA-binding protein (Park et al., 2013). This enzyme has two catalytic activities (Cheong et al., 2017); it can act as a peptidyl-prolyl cis-trans isomerase (PPIase) involved in remodeling protein structures or as a reductase that transfers electrons from one protein to another through its sequential reduction and oxidation. When OPDA is present and binds to CYP20-3, it forms a complex with serine acetyltransferase 1 (SAT1) and O-acetylserine (thiol) lyase B (OASTL-B) (Cheong et al., 2017; Liu and Park, 2021). Electron transfer to SAT1 stimulates sulfur assimilation leading to cysteine and methionine biosynthesis. Cysteine and methionine can be further metabolized into compounds involved in redox homeostasis or plant defense, such as glutathione or glucosinolates in arabidopsis (Takahashi, 2011). Thus, CYP20–3 is involved in controlling the balance between redox detoxification for carbohydrate biosynthesis and stress responses (Cheong et al., 2017).

CYP20–3 is constitutively present in chloroplast stroma and can be reduced by thioredoxins carrying electrons from the photosynthetic electron transport chain (pETC) (Dominguez-Solis et al., 2008; Cheong et al., 2017). Increasing atmospheric eCO_2_ level are predicted to influence electron flux through the pETC which may result in changes in electron flow to CYP20–3 and, thus, its interaction with OPDA and glutathione metabolism.

The Foyer-Halliwell-Asada (FHA) cycle is a series of connected redox steps that reduce the reactive oxygen species hydrogen peroxide (H_2_O_2_) to water using glutathione (Foyer and Noctor, 2011). Wounding plant leaves leads to rapid increases in reactive oxygen species (ROS), such as H_2_O_2_, at the wound site and systemically (Prasad et al., 2020; Fichman and Mittler, 2021). This increase in the oxidative state of the cell is both buffered and translated into downstream signaling through the FHA cycle, which links changes in redox metabolites to activation of phytohormone biosynthesis and plant resistance (Foyer and Noctor, 2013). Total and the oxidized-to-reduced ratios of metabolites in this pathway, particularly ascorbate and glutathione, reflect the cellular redox balance and, thus, are important signals of cellular stress levels. In particular, changes in these metabolites can activate salicylic acid (SA) or jasmonate pathways (Cheng et al., 2015; Han et al., 2013a, Han et al., 2013b); however, the subtleties distinguishing activation of one phytohormone pathway versus the other are not yet well understood (Noctor et al., 2024).

In arabidopsis, foliar levels of total glutathione and reduced NADPH were higher in plants grown at eCO_2_, which translated into higher SA levels (Mhamdi and Noctor, 2016). In contrast, in wounded arabidopis leaves, lower JA levels were seen in plants grown at eCO_2_ that likely reflected differences in NADP, reduced ascorbate (AsA) and total ascorbate (Asc) levels seen between plants grown in aCO_2_ or eCO_2_ environments (Martinez Henao et al., 2020). Thus, phytohormone levels of C3 plants are impacted by eCO_2_, however, the underlying mechanism is not well understood.

In general, both the SA-signaling pathway, leading to plant resistance against biotrophs, and the jasmonate-signaling pathway, leading to plant resistance against necrotrophic pathogens and chewing insect herbivores, may be affected by increasing atmospheric CO_2_ levels (Yang et al., 2015; Mhamdi and Noctor, 2016; Martinez Henao et al., 2020; Bazinet et al., 2022; Hou and Tsuda, 2022). In wounded arabidopsis grown at eCO_2_, jasmonate levels are attenuated but levels of the biosynthetic precursor OPDA are unchanged (Martinez Henao et al., 2020). In mechanically damaged leaves of arabidopsis grown at eCO_2_, the increased flux through the pETC may lead to higher reduction of CYP20-3, increasing its binding to wound-associated increases in OPDA and limiting OPDA flux into JA-Ile biosynthesis and, possibly, increasing glutathione biosynthesis. In this study, we investigate the role of CYP20–3 in regulating jasmonate biosynthesis in arabidopsis grown at eCO_2_ by comparing the wound responses in wildtype (WT) and *cyp20–3* knockout plants.

## Methods and materials

### Arabidopsis lines

*Arabidopsis thaliana* WT (Col-0) and *cyp20-3* (SALK_001615C) seeds were obtained from the Arabidopsis Biological Research Center. The zygosity of the T-DNA inserts in the *cyp20–3* SALK line were confirmed to be homozygous by polymerase chain reaction (PCR) using gene-specific primers and a left border primer complementary to the T-DNA insert (**Supplementary Table 1**). The absence of CYP20–3 gene expression and protein in the SALK line was verified by RNA-Seq and Western blot using an anti-CYP20–3 antibody (PhytoAB) (**Supplementary Figure 1**).

### Plant maintenance

Seeds were surface-sterilized by washing in 3% NaOCl for one min, 70% EtOH for one min and three rinses with sterile distilled water. Seeds were stratified in the dark for 3 days at 4 °C then sown in damp potting mix (Fafard Agro Mix G6) in individual pots (square (6.7 cm x 6.7 cm x 8.9 cm)) for the wounding time course experiments. Pots were placed in growth chambers (Conviron GEN 1000) with light and temperature settings programmed to simulate conditions in late May and early June in Montreal: 15 h light (175 μmol m^-2^ s^-1^) at 22 °C, light and temperature ramping over 3 h, 4 h dark at 18 °C, light and temperature ramping over 2 h. CO_2_ levels were maintained at 450 ± 20 ppm (ambient, aCO_2_) or 900 ± 50 ppm (elevated, eCO_2_) since the RCP8.5 model predicts that tropospheric CO_2_ levels will reach this level by 2100 (Lee et al., 2021). Pots were bottom-watered up to 3 times/week with dilute nitrogen: phosphorous: potassium fertilizer (3.4:3.4:3.4). When plants were at growth stage 3.9 (Boyes et al., 2001), they were used in wounding experiments.

### Arabidopsis wounding time-course experiment

Since CO_2_ levels affect plant developmental timing (Ward and Kelly, 2004), experiments were staggered by up to 3 days to ensure that plants were used at the same growth stage (stage 3.9 (Boyes et al., 2001)). Before the wounding time course, a plexiglass panel (61 cm (w) x 106 cm (h)) was placed snugly in the middle of the chamber between the wounded and unwounded plants to minimize volatile signaling between these treatments. Even though the plexiglass minimized the potential of volatile signaling, eCO_2_ levels in the chamber were not affected.

At the start of the growth cabinet ramping from light-to-dark, half of the plants were wounded. In each wounded plant, approximately 20**%** of the 7 largest leaves from each plant was wounded with a hole punch taking care not to damage the midvein (**Supplementary Table 4**). At specific time points after wounding, wounded and unwounded plants were harvested, immediately frozen in liquid nitrogen, and then stored at -80°C. For phytohormone analysis, rosettes were collected at 15 min, 30 min, 90 min and 5 hr after wounding. Rosettes for redox metabolite analysis were collected at 90 min after wounding. For gene expression analyses, 3 mid-sized leaves were harvested ensuring that these leaves had been damaged for the wounded plant samples. For qRT-PCR, these leaves were collected 15 min, 30 min, 90 min and 5 hr after wounding. For RNA-Seq, leaves were only collected at the 90 min timepoint. In addition, unwounded plants were harvested at time 0. The time course was temporally repeated 5 times with 1 biological replicate taken for the different analyses each time. Thus, the final replicates are n = 4 for phytohormones, n = 3 for redox metabolites, n = 4 for RNA-Seq and n = 5 for qRT-PCR.

In addition, wounded and unwounded rosettes were harvested to quantify plant biomass (n ≥ 9). The percentage dry weight removed by wounding was calculated as the difference between the average biomass of unwounded and wounded rosettes divided by the average biomass of unwounded samples.

### Phytohormones

Phytohormone quantification was conducted based on the protocol by Machado et al. (2013). Rosettes were lyophilized and finely ground. Approximately 20 mg plant sample were extracted in HPLC-grade methanol:water (70:30) containing deuterated phytohormone standards (40 ng/mL; D_6_-ABA, D_6_-JA, D_6_-JA-Ile D_6_-SA, D_5_-IAA). Following homogenization (Retsch MM 400, 10 min vibration at 30 hertz), samples were centrifuged to remove debris (20 min x 18,994 *g*, 20 °C). The supernatant was transferred to a new tube and evaporated to dryness in a speed vaccum (Labconco) at room temperature. Pellets were resuspended in HPLC-grade methanol:water (70:30) using an ultrasonic bath. Samples were recentrifuged (5 min x 18,994 *g*, room temperature) and the supernatant transferred to a fresh tube.

Phytohormone analysis was performed by ultrahigh performance liquid chromatography (UPLC, Waters Acquity) coupled to a mass spectrometer (Bruker Elite EvoQ triple-quadrupole). Compounds were separated by reverse phase chromatography on a Zorbax Eclipse XDB-C18 column (4.6 x 50 mm, 1.8 μm, Agilent). The mobile phase was held stationary for the first 30 sec at 5% acetonitrile (ACN) in water containing 0.1% formic acid. Over the next 10 sec, this was increased to 50% ACN, 0.1% formic acid and then to 100% ACN, 0.1% formic acid over the next 90 sec. After holding at 100% ACN, 0.1% formic acid for one minute, the mobile phase returned to initial conditions over 1 min. The flow rate was 400 µL/min and the column temperature was 42 °C.

Separated compounds were nebulized by electrospray ionization in the negative ion mode. The capillary voltage was set to 4500 eV. Cone, probe and nebulizer gases were set to 35 arbitrary units (a.u.)/350 °C, 60 a.u./475 °C, and 60 a.u., respectively. MS Data Review software (Bruker MS Workstation, vers. 8.2) was used for data processing. Phytohormones were identified based on the retention times and m/z transitions monitored (**Supplementary Table 2**). Phytohormone concentrations were calculated from the peak area of the phytohormone of interest relative to its deuterated standard divided by the dry weight of the extracted leaf material.

### Redox metabolites

Since JA-Ile levels were lower in wounded WT plants grown at eCO_2_ compared to aCO_2_ at 90 minutes after damage, this timepoint was selected for the analyses of redox metabolites. Oxidized and reduced forms of glutathione and ascorbate were measured using an enzymatic cycling method (Noctor and Mhamdi, 2022). Frozen rosette tissue was ground to a fine powder in liquid nitrogen using a mortar and pestle. Finely-ground tissue (100–150 mg) was extracted in 0.2 M HCl. After centrifugation (10 min x 10,000 *g*, 4 °C) and transfer of the supernatant to a new tube. Phosphate buffer (final concentration 33 mM, pH 5.6) was added and the pH adjusted to pH 5 by careful stepwise addition of 0.2 M NaOH to generate the neutralized extracts. For the measurement of reduced ascorbate (AsA), neutralized plant extract (40 μL) was added to wells containing phosphate buffer (final concentration 0.1 M, pH 5.6) and the absorbance at 265 nm measured. Ascorbate oxidase (0.2 U) was added followed by shaking (8 min) and measurement again at A_265_. The change in absorbance was used to calculate the concentration of reduced ascorbate (AsA) based on the molar extinction coefficient of 14 mM^-1^cm^-1^. Total ascorbate was measured by pre-treating aliquots of neutralized plant extract in the phosphate buffer (final concentration 0.112 M, pH 5.6) with dithiothreitol (final concentration 1 mM) for 30 min at room temperature followed by spectrophotometric measurement at A_265_. Oxidized ascorbate (DHA) was determined by subtracting AsA from total ascorbate.

For the measurement of total glutathione, neutralized plant extract (10 µL) was added to wells containing a reaction mixture which had a final concentration after the addition of sample of 0.1 M phosphate buffer (pH 7.5) with 5 mM EDTA, 0.5 mM NADPH and 0.6 mM 5,5 dithiobis 2-nitro-benzoic acid. Glutathione reductase (0.2 units) was added to initiate the reaction. The increase in absorbance at 412 nm was monitored for 5 min at 20 s intervals. The slope of the linear part of the curve (A_412_ vs time) was used to calculate glutathione concentration from a standard curve of glutathione (0 to 0.4 nmol, 5 concentrations). For the measurement of oxidized glutathione (GSSG), aliquots of neutralized extracts as well as GSSG standards (0 to 160 pmoles, 5 concentrations) were incubated with 2-vinylpyridine for 30 min to precipitate reduced glutathione (GSH). Following centrifugation twice (10,000 x *g*, 15 min, room temperature) with the sample transferred to a new tube between centrifugation steps, GSSG levels were measured spectrophotometrically as outlined above. The amount of GSH was calculated as total glutathione minus (2 x GSSG).

### Transcriptomics

#### RNA extraction

Arabidopsis rosettes were finely ground in liquid nitrogen. Total RNA was extracted using the RNeasy Plant Mini Kit (Qiagen) according to the manufacturer’s instructions. The quality (absorbance ratios A_260_/A_280_ and A_260_/A_230_) and concentration of the RNA extracts was determined by NanoDrop spectrophotometry. In addition, the RNA quality was assessed after separation on a 1% agarose gel containing 1% bleach including SYBR Safe DNA Gel Stain (Invitrogen) to enable visualization with a Gel Doc imaging system (Bio-Rad) (Aranda et al., 2012).

#### RNA-Sequencing and analysis

Total RNA (100 ng/µL) was brought to Genome Québec for polyA-enriched library preparation and 100 base paired-end RNA-sequencing by NovaSeq PE100bp (Illumina). After removal of adapter sequences, *Fastp* was used to remove low quality bases at the ends of the raw reads and reads shorter than 25 bases (Chen et al., 2018). The quality of the raw reads was evaluated using FastQC (Andrews et al., 2010). The processed reads were mapped to the *A. thaliana* reference genome (assembly 10.1 downloaded from NCBI) using STAR (Dobin et al., 2013). The aligned reads, in Binary Alignment/Map (BAM) files, were input into *featureCounts* to quantify the strand-specific read counts per gene (Liao et al., 2013). The raw read data (FASTQ) has been deposited in the NCBI Sequence Read Archive (Bioproject ID #PRJNA1332053).

The resulting gene counts were uploaded to ExpressAnalyst Pro for normalization by Log_2_ counts per million transformation and DESeq2 conducted to determine differentially expressed genes (Liu et al., 2023; Love et al., 2014). The criteria to determine wound-induced differentially expressed genes in WT plants grown at aCO_2_ or wound- or SA-responsive genes differentially expressed in WT or *cyp20–3* plants grown at aCO_2_ was a Log_2_ fold-change (Log_2_FC) of ≥ 2 (induced) or ≤ -2 (repressed) and an adjusted p value of ≤ 0.05. Once WT wound-induced genes in plants grown at aCO_2_ were identified, differences between WT or *cyp* genotypes grown at aCO_2_ or eCO_2_ was determined by DESeq2 with a p ≤ 0.05.

Data were visualized using MetaboAnalyst or ExpressAnalyst platforms (Liu et al., 2023; Pang et al., 2024). KEGG terms were identified using https://www.genome.jp/kegg/. Gene ontology (GO) terms were identified by GO Enrichment Analysis (Ashburner et al., 2000; Thomas et al., 2022; Gene Ontology Consortium, 2026).

### Quantitative reverse transcription-polymerase chain reaction

Gradient PCR was used to optimize the annealing temperature of gene-specific primers, identified from the literature or designed with primerBLAST (**Supplementary Table 1**). The amplification efficiency for each primer pair was determined by qRT-PCR (Mx3000) by performing standard curves with 8 x 10-fold dilutions of the PCR amplicon as the template (0.05 fg to 0.5 ng) using the following thermocycling program: 95 °C for 1 minute; 40 cycles of 95 °C for 15 seconds, annealing temperature for 30 seconds, 72 °C for 10 seconds, measurement of fluorescence at the end of each cycle; melt from 60 °C to 95 °C. Annealing temperatures and primer concentrations were adjusted to ensure 90-110% amplification efficiencies (Taylor et al., 2019). Amplicons were cloned into pUCm-T vectors (BioBasic) and sent to Genome Québec for Sanger sequencing to confirm the target sequences.

From plant total RNA extracted as described above, RNA (1 µg) was treated with DNase and reverse transcribed to cDNA using a Quantitect reverse transcription kit (Qiagen) according to the manufacturer’s instructions. PCR with primers spanning an intronic region of *IRX12/AtLMCO4* was used to confirm the absence of genomic DNA in the cDNA (Weech et al., 2008).

qRT-PCR reactions were conducted in white 96-well qPCR plates. Each well contained 1 x Luna Universal qPCR Master Mix (New England Biolabs), gene-specific forward and reverse primers (200–350 nM), 5 ng of cDNA, and nuclease-free water to a final volume of 10 µL. Each sample was spotted in triplicate. Non-template controls (NTCs) and inter-run calibrators (IRCs) consisting of pooled cDNA were included on each plate. Inter-run calibration was performed using the MxPro software to adjust the amplification thresholds so that the quantification cycle (Cq) values are the same for all IRC reactions for each gene.

Amplification was performed using the following conditions: 95 °C for 1 minute; 40 cycles of 95 °C for 15 seconds, annealing temperature for 30 seconds, 72 °C for 10 seconds, measurement of fluorescence at the end of each cycle; melt from 60 °C to 95 °C. Two technical plate replicates were performed.

The initial template concentration (R0) for each gene was calculated as R0 = 1/(1+E)^Cq^, where E is the amplification efficiency for the gene-specific primer pair and Cq is the quantification cycle (Zhao and Fernald, 2005). Relative gene expression levels were normalized to the geometric mean of 3 reference genes (*AtCBP20*, *AtACT2* and *AtTIP41*) (Vandesompele et al., 2002). The stable expression of these reference genes was verified using geNorm in the web-based tool RefFinder and gene stability value were below 0.5 (Xie et al., 2023).

### Statistics

Except for the RNA-Seq data, in general, outliers were detected using the maximal normed residual test (Grubb’s test) and excluded from analysis (Stefansky, 1971). Data were analyzed by analysis of variance (ANOVA) using SPSS (vers. 29, IBM) followed by Tukey honest significant difference (HSD) *post-hoc* test. If a significant interaction effect was observed, the results were further teased apart by a 2- or 1-factor ANOVA.

To determine if genotype or CO_2_ affected plant biomass, rosette dry weight was analyzed by 2-factor analysis-of-variance (ANOVA) (Factors: genotype, CO_2_). To determine the amount of biomass removed by wounding, the dry weight of wounded rosettes was subtracted from dry weights of whole rosettes and % removed compared by 2-factor ANOVA (Factors: genotype, CO_2_).

Phytohormone levels in undamaged and damaged plants were compared by 2-factor ANOVA (Factors: wounding, time) to determine if there are changes in response to wounding. Plants were then separated into unwounded or wounded and separately analyzed by 3-factor ANOVA (Factors: genotype, CO_2_, time). Data that violated Levene’s test of homogeneity were normalized by log_10_ transformation.

Redox metabolites were analyzed by 3-way ANOVA (Factors: CO_2_, genotype, wounding). Data that violated Levene’s test of homogeneity were normalized by log_10_ transformation.

Statistical analyses and visualization for RNA-Seq data is explained in the RNA-Seq section above. Gene expression measured by qRT-PCR was analyzed separated in unwounded and wounded plants by 3-factor analysis-of-variance (Factors: CO_2_ level, genotype, time). Data that violated Levene’s test of homogeneity were normalized by log_2_ transformation.

## Results

### Plant biomass

The *cyp20–3* arabidopsis plants were smaller (38%) than WT plants (**Supplementary Figure 2**; **Supplementary Table 3**) (F_(1,68)_ = 45.03, p < 0.001). Because of the known effect of CO_2_ on plant development (Ward and Kelly, 2004), the wounding experiment on plants grown at aCO_2_ or eCO_2_ was staggered, up to 3 days, to ensure they were at the same growth stage. When plants were at the 3.9 growth stage (Boyes et al., 2001), approximately 20% of rosette biomass was removed by mechanical wounding and this was consistent between temporal replicates (**Supplementary Figure 2**; **Supplementary Table 4**). The percent tissue removed by wounding was the same for both WT and mutant plants at both CO_2_ levels (**Supplementary Table 4**).

### Phytohormones

**Figure 1:**
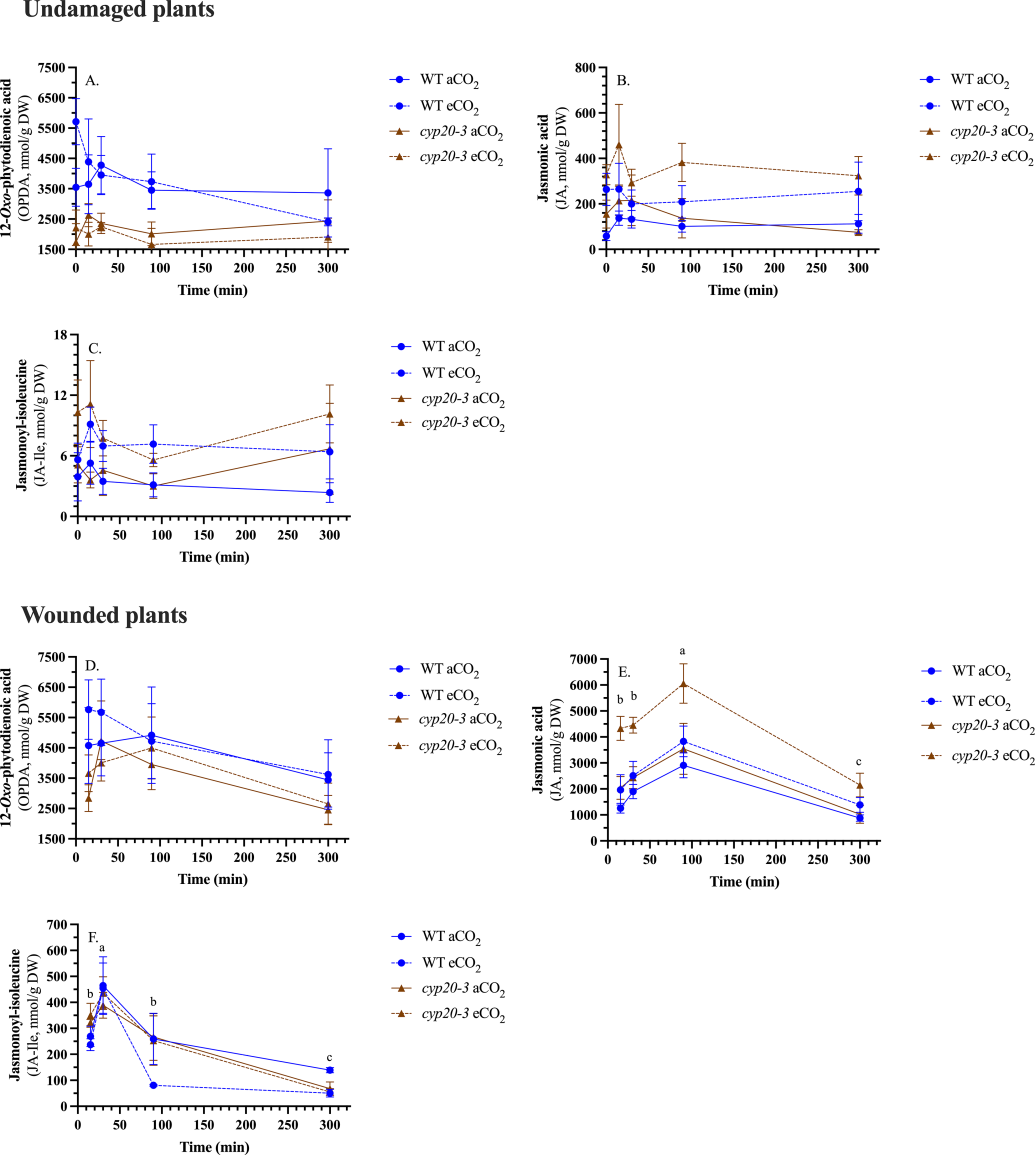
CO_2_ and genotype affect jasmonate levels in *Arabidopsis thaliana* rosettes. Wildtype (WT) and *cyp20–3* plants were grown at ambient CO_2_ (aCO_2_, 450 ppm) or elevated CO_2_ (eCO_2_, 900 ppm). Rosettes were either left undamaged or wounded when plants reached growth stage 3.9 (Boyes et al., 2001). Jasmonates were measured by ultrahigh performance liquid chromatography-mass spectrometry. Jasmonate levels in undamaged or damaged plants were analyzed by 3-factor analysis-of-variance (ANOVA) (Factors: CO_2_, genotype, time) followed by Tukey HSD *post-hoc* test to identify significant differences (**Supplementary Table 5**). Undamaged plants **(A)** 12-*oxo*-phytodienoic acid levels are higher in WT plants, **(B)** jasmonic acid (JA) levels are higher in *cyp20–3* plants and in plants grown at eCO_2_, and **(C)** jasmonoyl-isoleucine (JA-Ile) levels are higher at eCO_2_. Wounded plants **(D)** OPDA levels are higher in WT plants, **(E)** JA levels are higher in the *cyp20–3* mutant grown at eCO_2_, and **(F)** JA-Ile levels are lower at eCO_2_. Temporal changes in JA and JA-Ile levels after wounding are denoted by alphabetical letters. Data points represent the mean ± standard error.

In undamaged arabidopsis rosette leaves, jasmonate levels were basal (**Figure 1**; **Supplementary Table 5**). Of interest, OPDA, the precursor to JA and JA-Ile, was higher in WT plants than *cyp20-3*, while JA was higher in the *cyp20–3* mutant (**Figures 1A, B**). JA and JA-Ile levels in undamaged plants were minimally higher in plants grown at eCO_2_ compared to aCO_2_ (**Figures 1B, C**).

Rosette OPDA levels did not change in response to wounding and remained higher in WT plants compared to the *cyp20–3* mutant (**Supplementary Table 5**) (**Figure 1D**). In contrast, JA and JA-Ile levels increased rapidly, over 50 times levels in undamaged plants, in the first 15 min after wounding (**Figures 1E, F**). JA levels were higher in the *cyp20–3* mutant grown at eCO_2_ than in the other plants (WT-aCO_2_, WT-eCO_2_ or *cyp20-3*-aCO_2_). In contrast, JA-Ile levels were higher in plants grown at aCO_2_ compared to those at eCO_2_, which is particularly evident in WT plants 90 min after wounding.

**Figure 2:**
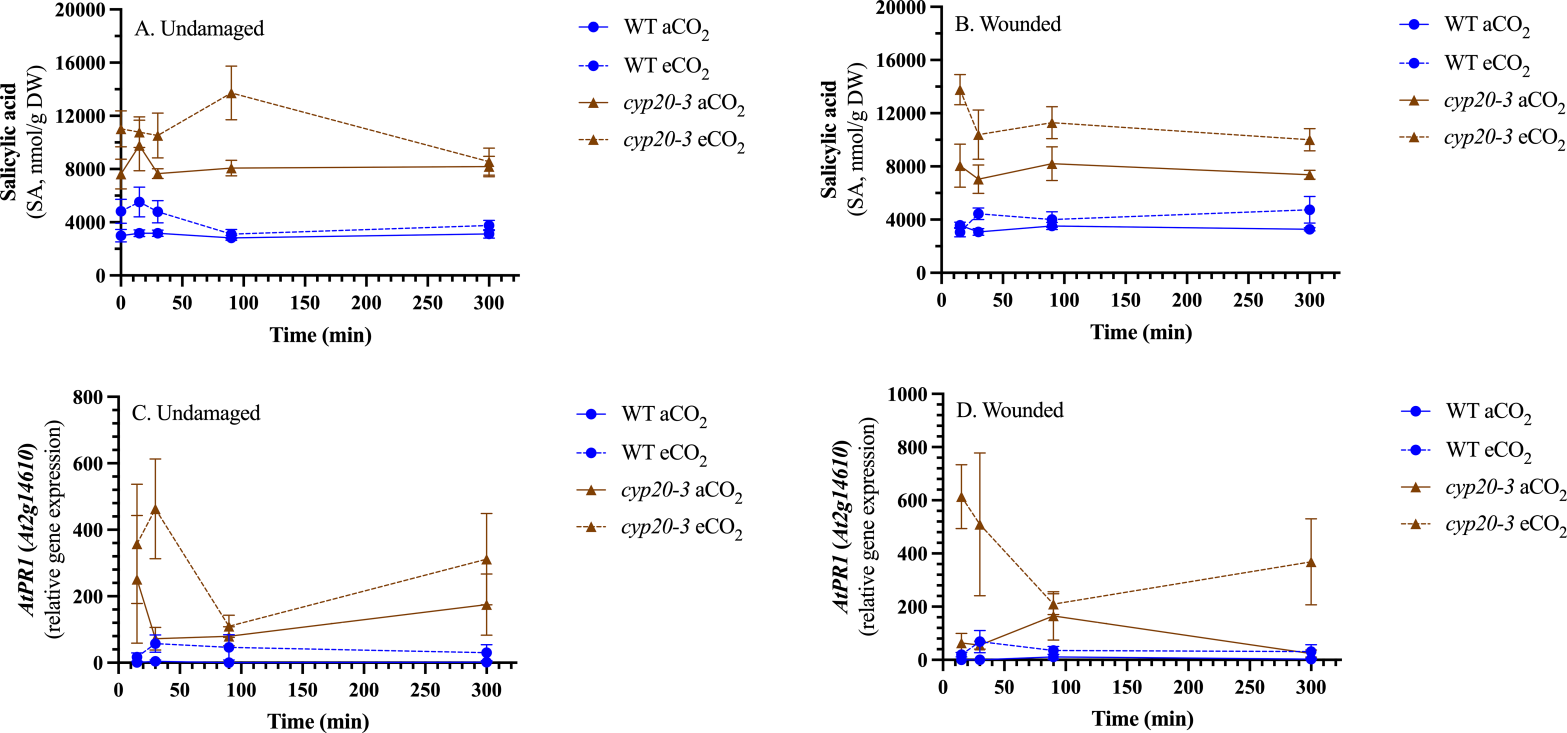
CO_2_ and genotype affect salicylic acid levels in *Arabidopsis thaliana* rosettes. Wildtype (WT) and *cyp20–3* plants were grown at ambient CO_2_ (aCO_2_, 450 ppm) and elevated CO_2_ (eCO_2_, 900 ppm). Rosettes were either left undamaged or wounded when plants reached growth stage 3.9 (Boyes et al., 2001). Salicylic acid (SA) was measured by ultrahigh performance liquid chromatography-mass spectrometry. Phytohormone levels in undamaged or damaged plants analyzed by 3-factor analysis-of-variance (ANOVA) (Factors: CO_2_, genotype, time) followed by Tukey HSD *post-hoc* test to identify significant differences (**Supplementary Table 5**). Expression of a SA-responsive gene, *AtPR1*, was measured by quantitative reverse transcription-polymerase chain reaction (qRT-PCR). After normalization to the geometric mean of three reference genes, *AtPR1* expression was analyzed by 3-factor analysis-of-variance (ANOVA) (Factors: CO_2_, genotype, time) followed by Tukey HSD *post-hoc* test to identify significant differences (**Supplementary Table 6**). Salicylic acid levels are higher at eCO_2_ and in the *cyp20–3* mutant in **(A)** undamaged, and **(B)** wounded plants. Expression of *AtPR1* is higher at eCO_2_ and in the *cyp20–3* mutant in **(C)** undamaged, and **(D)** wounded plants. Data points represent the mean ± standard error.

To assess the potential role of phytohormone crosstalk in modulating jasmonate signaling, levels of SA and ABA were measured (**Figure 2**; **Supplementary Figure 3**). In both undamaged and damaged plants, SA levels were on average 1.4 x higher in plants grown at eCO_2_ compared to aCO_2_ (**Supplementary Table 5**). SA levels were also twice as high in the *cyp20–3* mutant compared to WT plants and this was reflected in the expression of the SA-responsive gene *AtPR1* (At2g14610). In undamaged rosettes, ABA levels were lowest in WT grown at eCO_2_ compared to the other plants (WT-aCO_2_, *cyp20-3*-aCO_2_ or *cyp20-3*-eCO_2_)(**Supplementary Table 5**). In wounded plants, ABA levels rose slightly at 90 min and were higher in *cyp20–3* mutant compared to WT plants.

### Redox metabolites

At 90 minutes after damage, foliar rosettes were taken for redox metabolite and transcriptomic analyses. This timepoint represents a clear point where JA-Ile levels in wounded WT plants grown at eCO_2_ are lower than the other plants (**Figure 1F**) (**Supplementary Table 5**).

**Figure 3:**
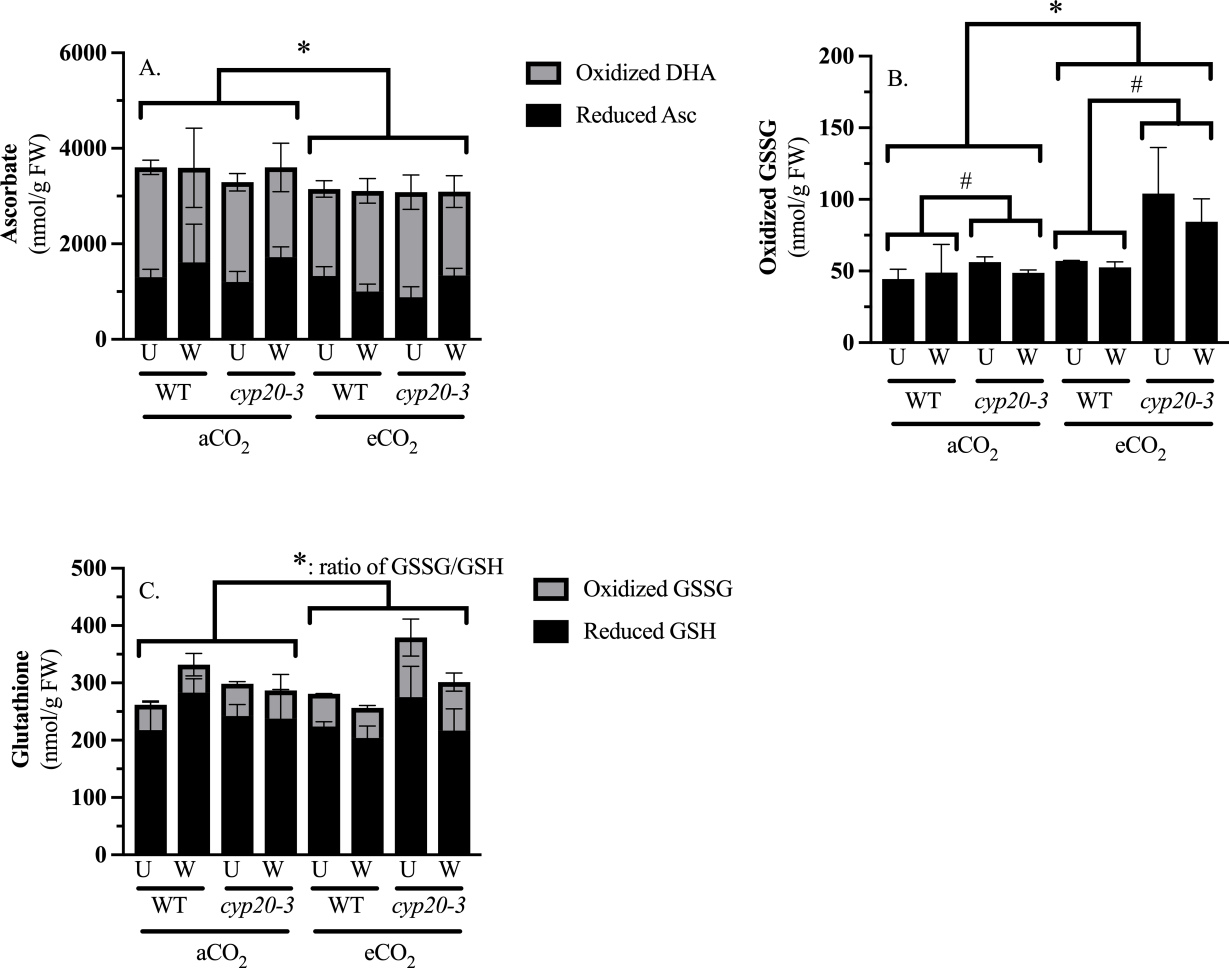
CO_2_ and genotype affect redox metabolite levels in *Arabidopsis thaliana* rosettes. Wildtype (WT) and *cyp20–3* plants were grown at ambient CO_2_ (aCO_2_, 450 ppm) or elevated CO_2_ (eCO_2_, 900 ppm). Rosettes were either left undamaged or wounded when plants reached growth stage 3.9 (Boyes et al., 2001). Levels of redox metabolites were measured through spectrophotometric assays and analyzed by 3-factor analysis-of-variance (Factors: CO_2_, genotype, wounding) followed by Tukey HSD *post-hoc* test to identify significant differences (**Supplementary Table 7**). **(A)** Lower total ascorbate levels are observed in plants grown at eCO_2,_ **(B)** Higher oxidized glutathione (GSSG) levels are observed in plants grown at eCO_2_ and in *cyp20–3* mutants, and **(C)** The ratio of GSSG-to-reduced glutathione (GSH) is higher in plants grown at eCO_2_. Bars represent the mean ± standard error. An asterisk represents differences due to CO_2_ levels. A number sign represents genotype differences.

Levels of intermediates in the FHA cycle, particularly ascorbate and glutathione and their reduced or oxidized forms, are indicators of the cellular redox state and oxidative stress (Foyer and Noctor, 2011). At 90 minutes after wounding, levels of redox metabolites are responding to changing oxidative stress to reduce levels of reactive oxygen species such as H_2_O_2_ and are acting as signaling molecules leading to phytohormone biosynthesis, both jasmonates and SA (Han et al., 2013a, Mhamdi et al., 2010). At this timepoint, wounding did not affect levels or oxidation state of ascorbate or glutathione (**Supplementary Table 7**). Total ascorbate levels were lower at eCO_2_, but CO_2_ levels did not affect the ratio of oxidized (DHA)-to-reduced (Asc) ascorbate (**Figure 3**).

CO_2_ affected glutathione in the opposite way. Oxidized GSSG levels were higher in the *cyp20–3* mutant plant, with this distinction more marked in plants grown at eCO_2_, as well as in arabidopsis grown at eCO_2_ (**Figure 3B**). This translated into a higher ratio of GSSG-to-GSH levels in plants grown at eCO_2_. (**Figure 3C**).

### Gene expression

After removal of adaptors and low-quality reads, an average of 43.7 million 100 bp reads per sample with a Pfred score above 20 was obtained (**Supplementary Table 8**). These high-quality reads were aligned to the arabidopsis TAIR10 genome with an average mapping efficiency of 95.0% uniquely mapped reads (**Supplementary Table 9**).

**Figure 4:**
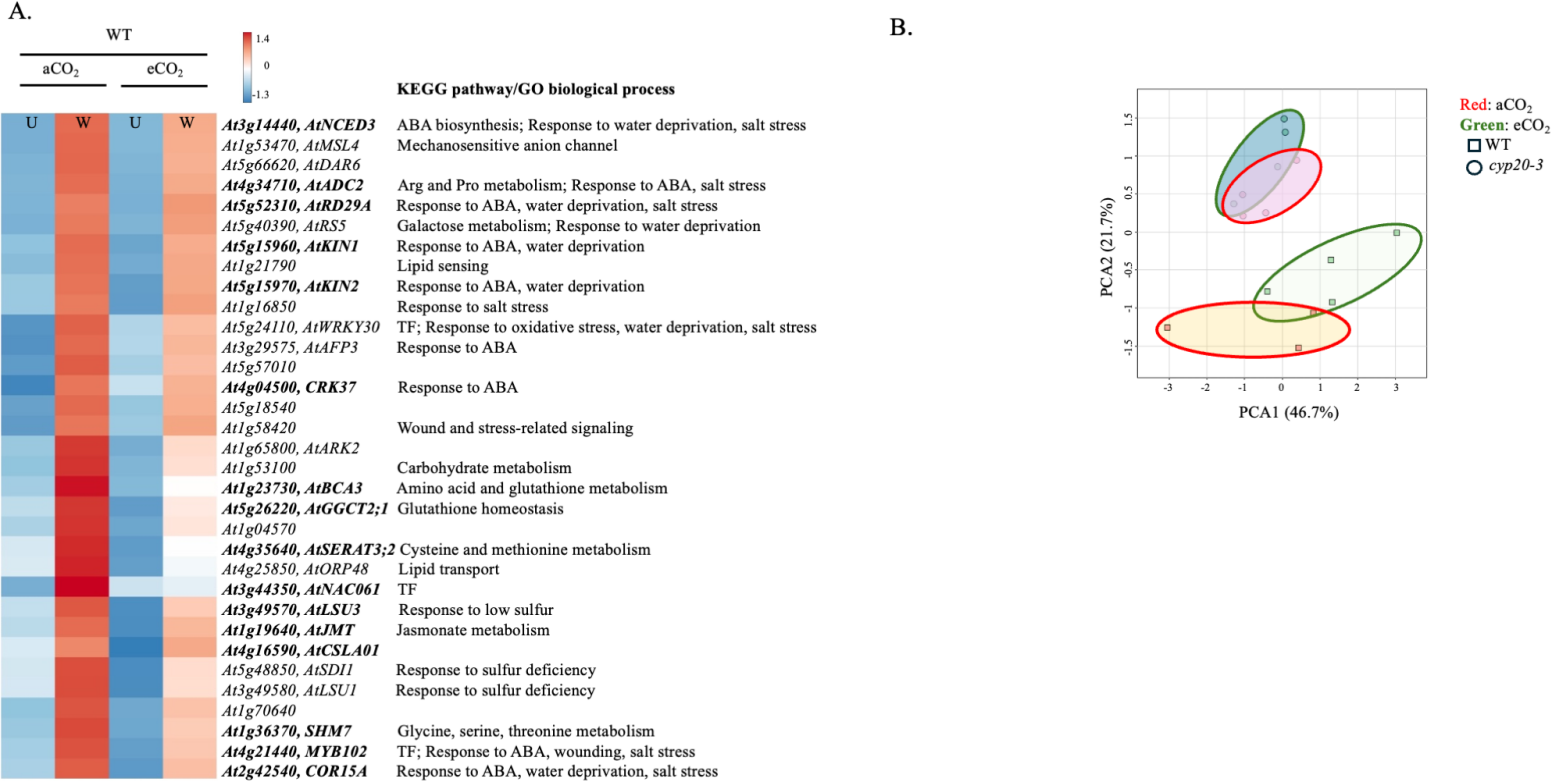
Wound-induced gene expression in WT *Arabidopsis thaliana* is attenuated in plants grown at eCO_2_. Wildtype (WT) arabidopsis were grown at ambient CO_2_ (aCO_2_, 450 ppm) or elevated CO_2_ (eCO_2_, 900 ppm). Rosettes were either left undamaged (U) or wounded (W) when plants reached growth stage 3.9 (Boyes et al., 2001). Total RNA was extracted for RNA-Seq (**Supplementary Table 8**). Wound-induced genes in WT plants grown at aCO_2_ (log_2_FC ≥ 2, ≤ -2, padj ≤ 0.05) were identified. From this list, genes differentially expressed between wounded WT plants grown at aCO_2_ and eCO_2_ were identified by DESeq2. **(A)** Heat map of genes downregulated in wounded WT grown at eCO_2_ compared to aCO_2_. KEGG and gene ontology (GO) terms were identified using www.genome.jp/kegg/ and GO enrichment analysis (Ashburner et al., 2000; Thomas et al., 2022; The Gene Ontology Consortium, 2026), respectively. Wound- and/or jasmonate-responsive genes are highlighted in bold. **(B)** Principal component analysis comparing the expression of these downregulated genes in WT (square) and *cyp20-3* (circle) plants grown at aCO_2_ (red) or eCO_2_ (green). Abbreviations: ABA, abscisic acid; TF, transcription factor.

In line with the lower JA-Ile levels in wounded WT plants grown at eCO_2_ (**Figure 1F**), a number of wound- or jasmonate-responsive genes, such as *AtBCA3, AtGGCT2, AtLSU3, AtSERAT3, AtNAC061, AtMYB102, AtSHM7, AtKIN1, AtNCED3, AtADC2, AtCRK37, AtJMT, AtKIN2, AtCSLA1*, and *AtRD29A*, had lower expression in wounded WT arabidopsis grown at eCO_2_ compared to aCO_2_ (**Figure 4A**) (Seo et al., 2001; Perez-Amador et al., 2002; Denekamp and Smeekens, 2003; Hossain et al., 2011; Hu et al., 2013; Choi et al., 2014; Thatcher et al., 2015; Hieno et al., 2019; Zander et al., 2020; Apodiakou and Hoefgen, 2023; Canales et al., 2023; Kaur et al., 2024). This eCO_2_-distinction seen in WT plants was disrupted in the *cyp20–3* mutant (**Figure 4B**).

**Figure 5:**
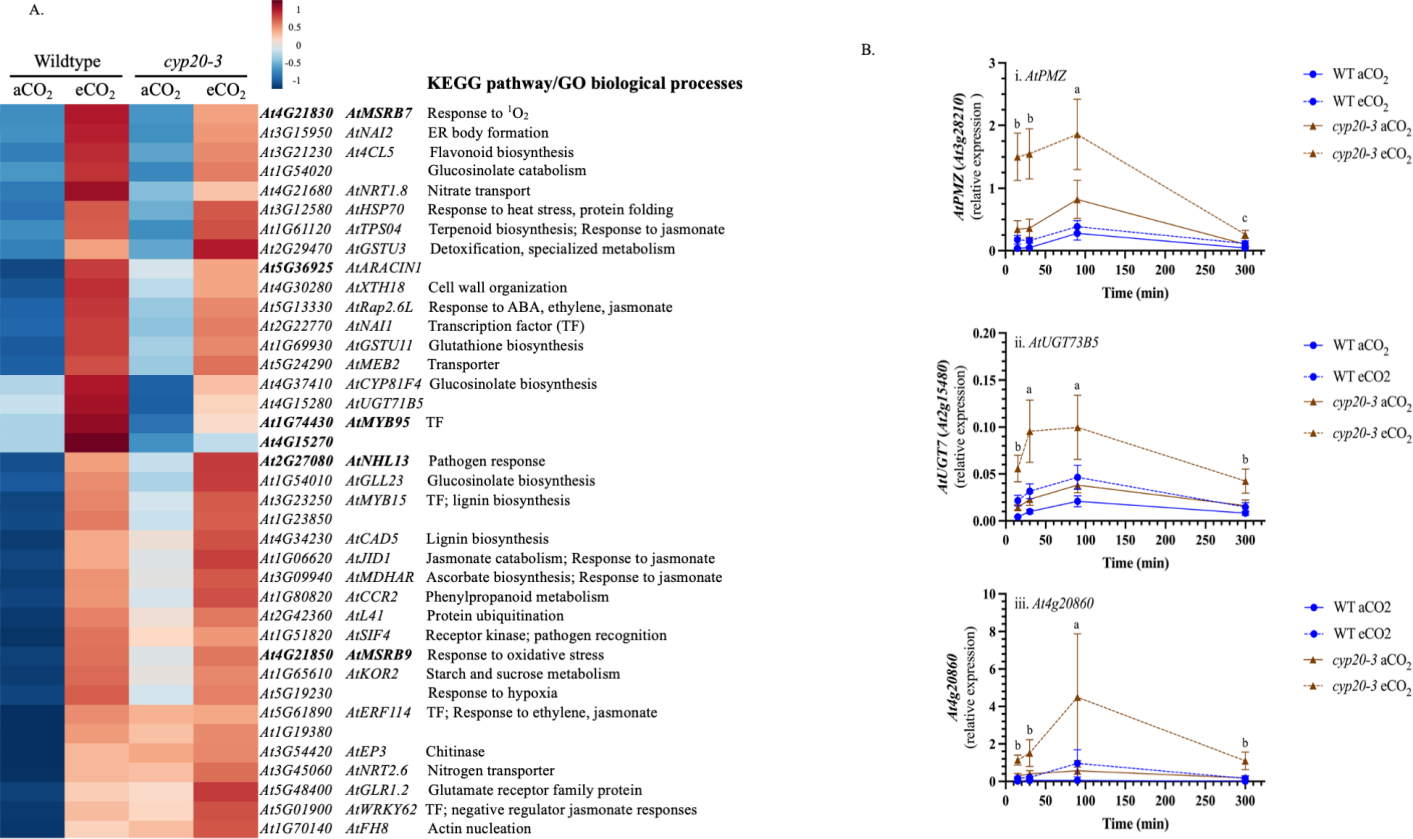
Wound-induced gene expression increased in WT *Arabidopsis thaliana* plants grown at eCO_2_. Wildtype (WT) or *cyp20–3* arabidopsis were grown at ambient CO_2_ (aCO_2_, 450 ppm) or elevated CO_2_ (eCO_2_, 900 ppm). Rosettes were either left undamaged or wounded when plants reached growth stage 3.9 (Boyes et al., 2001). Total RNA was extracted for RNA-Seq (**Supplementary Table 8**). From a list of wound-induced genes in WT plants grown at aCO_2_ (log FC ≥ 2, ≤ -2, padj ≤ 0.05) identified using DESeq2, differentially expressed genes between WT plants grown at aCO_2_ and eCO_2_ were determined. **(A)** Heat map of genes expressed at higher levels in wounded WT grown at eCO_2_ compared to aCO_2_. KEGG and gene ontology (GO) terms were identified using www.genome.jp/kegg/ and GO enrichment analysis (Ashburner et al., 2000; Thomas et al., 2022; The Gene Ontology Consortium, 2026), respectively. Oxidative stress-responsive genes are highlighted in bold. **(B)** Expression of jasmonate-independent, wound-responsive genes were measured by qRT-PCR. After normalization to the geometric mean of three reference genes, target gene expression was analyzed by 3-factor analysis-of-variance (ANOVA) (Factors: CO_2_, genotype, time) followed by Tukey HSD *post-hoc* test to identify significant differences (**Supplementary Table 6**). i) *AtPMZ* (At3g28210), ii) *AtUGT73B5* (*At2g15480*), and iii) *At4g20860*. Gene expression levels are higher in wounded plants at eCO_2_ and in the *cyp20–3* mutant (**Supplementary Table 6**). Data points represent the mean ± standard error. Temporal changes in wound-induced gene expression levels are denoted by alphabetical letters. Abbreviations: TF, transcription factor.

Some genes showed elevated expression in WT plants grown at eCO_2_ compared to aCO_2_ (**Figure 5A**); in this case, an enrichment of oxidative-stress related genes, such as *AtMYB95, AtAGO4, At4g15270, AtMSRB7, AtMSRB9* and *At5g36925* (Dos Santos et al., 2005; Mhamdi et al., 2010; Inzé et al., 2012; Waese and Provart, 2016) was observed. Of note, *AtMDHAR* which encodes monodehydroascorbate reductase which reduces monodehydroascorbate to AsA in the FHA cycle was upregulated in wounded WT plants grown at eCO_2_. *AtPMZ* (*At3g28210*)*, AtUGT73B5* (*At2g15480*) and *At4g20860*, genes identified as being jasmonate-independent, wound-responsive genes (Mekkaoui et al., 2025) also showed higher expression at eCO_2_ and in the *cyp20–3* mutant, supporting our observations of increased oxidative stress gene expression in wounded plants grown at eCO_2_ (**Figure 5B**) (**Supplementary Table 6**).

Of particular interest to OPDA metabolism, *AtJID1* was wound-induced and upregulated at eCO_2_ (**Figure 5A**). *AtJID1* encodes a 2-oxoglutarate/Fe(II)-dependent dioxygenase that modifies OPDA, limiting wound-induced accumulation of OPDA, JA and JA-Ile when overexpressed (Yi et al., 2023). Thus, although OPDA levels were unaffected by the CO_2_ level, OPDA catabolism initiated by JID1 may be increased at eCO_2_ which would limit JA and JA-Ile biosynthesis.

## Discussion

**Figure 6:**
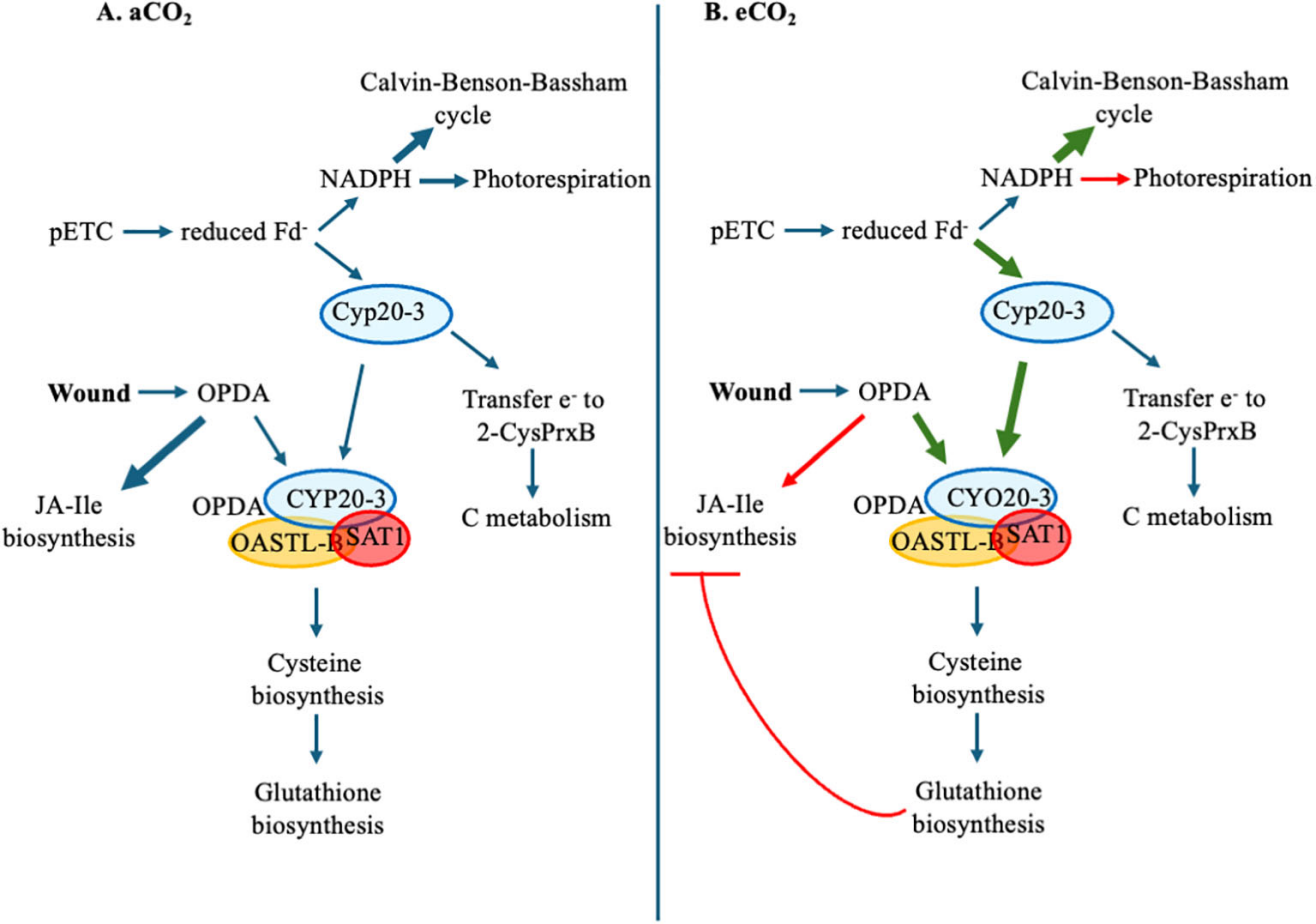
Model of CYP20-3-mediated suppression of the wound-induced jasmonate burst in C3 plants grown at eCO_2_. Increased atmospheric CO_2_ favours the carboxylation reaction of ribulose 1, 5-bisphosphate, enhancing the Calvin-Benson-Bassham cycle and reducing photorespiration. This could increase the availability of electrons from the photosynthetic electron chain to be passed to CYP20-3. In the presence of wound-associated 12-*oxo*-phytodienoic acid (OPDA), a complex is formed between CYP20-3-OPDA-SAT1-OASTL-B that stimulates cysteine biosynthesis. Cysteine is a residue in the tripeptide glutathione and increased levels of glutathione may dampen jasmonate biosynthesis. **(A)** aCO_2_. **(B)** eCO_2_. Green and red arrows represents processes enhanced or decreased at eCO_2_. Thickness of arrows represent the proposed strength into different pathways. aCO_2_, ambient CO_2_; C, carbon; eCO_2_, elevated CO_2_; Fd, ferredoxin; JA-Ile, jasmonoyl-isoleucine; NADPH, nicotinamide adenine dinucleotide phosphate; OASTL-B *O*-acetylserine(thiol)lyase-B; OPDA, 12-*oxo*-phytodienoic acid; pETC, photosynthetic electron transport chain; SAT1, *S*-adenosylmethionine acetyltransferase 1.

The wound-induced increase in jasmonates is attenuated in C3 plants grown at eCO_2_ which may lead to lower plant resistance against necrotrophic pathogens and chewing insect herbivores under future predicted environmental conditions (Bazinet et al., 2022 and references therein). Levels of JA-Ile are lower in wounded rosette leaves of arabidopsis grown at eCO_2_ compared to those grown at aCO_2_, but this CO_2_-dependent difference is not seen in the levels of their precursor OPDA, suggesting that OPDA may be blocked from continuing into jasmonate biosynthesis (**Figures 1D, F**). Our results suggest that CYP20–3 affects the wound-induced jasmonate biosynthesis in arabidopsis grown at eCO_2_ through two related mechanisms. First, OPDA binding to CYP20–3 limits OPDA flux into JA-Ile biosynthesis. In addition, this interaction promotes the production of glutathione, which buffers changes in cellular redox state to mute signaling leading to jasmonate biosynthesis (**Figure 6**).

Upon reduction by electrons from the pETC, CYP20–3 binds to OPDA forming a complex with SAT1 and OASTL-B (CYP20-3-OPDA-SAT1-OASTL-B), potentially limiting OPDA flux into jasmonate biosynthesis which was confirmed in our study (Liu and Park, 2021); OPDA levels are lower in wounded *cyp20–3* plants with higher JA levels observed in *cyp20–3* grown at eCO_2_ (**Figures 1D, E**). This suggests that in arabidopsis grown at eCO_2_ increased binding of CYP20–3 to OPDA blocks flux into downstream jasmonate biosynthesis. Another possible point of regulation of OPDA into JA-Ile production is through the action of the 2-oxoglutarate/Fe^(II)^-dependent dioxygenase JID1 that modifies OPDA, limiting wound-induced accumulation of OPDA, JA and JA-Ile when overexpressed in arabidopsis (Yi et al., 2023). We observed that *AtJID1* is wound-induced and upregulated at eCO_2_ (**Figure 5A**), suggesting that the enzyme may limit OPDA conversion to JA-Ile, but this needs to be experimentally validated. It is also noteworthy that JA-Ile, but not JA, levels are lower in WT plants grown at eCO_2_ (**Figure 1F**), suggesting there is likely another block in JA-Ile biosynthesis between JA and JA-Ile.

Binding of OPDA to form the CYP20-3-OPDA-SAT1-OASTL-B complex not only removes OPDA from jasmonate biosynthesis but promotes cysteine biosynthesis leading to the production of the redox metabolite glutathione (Cheong et al., 2017; Liu and Park, 2021). Cysteine is needed for the biosynthesis of the tripepeptide redox metabolite glutathione (glutamate-cysteine-glycine) (Liu and Park, 2021; Takahashi, 2011). We did not see a change in reduced or total glutathione levels, but GSSG and the ratio of GSSG-to-GSH are higher in plants grown at eCO_2_ suggesting that eCO_2_ leads to a more oxidized cellular environment (**Figures 3B, C**). In addition, *cyp20–3* plants had higher GSSG levels than WT, particularly when grown at eCO_2_ (**Figure 3B**), suggesting that CYP20-3-associated glutathione production leads to a more reduced cellular environment.

Changes in the oxidation state or levels of these redox metabolites provide information on the cellular redox state and are involved in the regulation of various signaling pathways, including phytohormone biosynthesis (Foyer and Noctor, 2011). At 90 minutes post-wounding, a strong CO_2_-dependent difference in foliar cellular redox metabolites is noted (**Figure 3**); specifically, lower total ascorbate and higher oxidized glutathione (GSSG) levels in arabidopsis grown at eCO_2_ compared to aCO_2_. This increased oxidative environment is reflected in wound-induced, oxidative stress genes upregulated in both WT and *cyp20–3* plants (**Figure 5**).

Plant stresses can perturbate cellular redox status leading to changes in phytohormone SA and JA levels, that, in turn, affects redox metabolite levels and ratios (Foyer and Noctor, 2011). In arabidopsis, higher SA levels are often correlated with increased total and oxidized glutathione levels (Han et al., 2013a), which suggests that genotype- and CO_2_-dependent increases in GSSG may manifest in higher observed levels of foliar SA and expression of the SA-responsive gene *AtPR1* (**Figures 2**, **3B, C**). The crosstalk between jasmonate and SA signaling in plant stress responses is well established (Caarls et al., 2015; Yang et al., 2015; Aerts et al., 2021; Hou and Tsuda, 2022). Thus, the enhanced foliar SA levels may contribute to the lower wound-associated jasmonate levels in arabidopsis grown at eCO_2_ (**Figures 1F**, **4A, B**). As well, higher expression of the SA-responsive *AtWRKY62* is noted in wounded plants grown at eCO_2_ compared to those grown at aCO_2_ (**Figure 5A**). This transcription factor interferes with jasmonate signaling and the induction of jasmonate-responsive gene expression (Mao et al., 2007). However, both jasmonate and SA levels were higher in *cyp20–3* than in WT at eCO_2_, suggesting that SA-mediated antagonism of jasmonate biosynthesis cannot fully explain the eCO_2_ attenuation of wound-associated JA-Ile.

Changes in cellular reduced GSH and AsA levels are thought to positively stimulate jasmonate biosynthesis and/or signaling (Han et al., 2013b; Suza et al., 2010). Even in the absence of wounding, oxidative stress induced by increased production of H_2_O_2_ can activate jasmonate-responsive gene expression through a GSH-dependent mechanism (Han et al., 2013b). Treatment of arabidopsis with AsA or plants with elevated AsA levels have increased foliar jasmonate levels (Nepal et al., 2019; Bulley et al., 2021). However, wounding- or jasmonate treatment have also been seen to increase foliar ascorbate levels in some plant species (Suza et al., 2010). In our study, ascorbate levels were reduced in arabidopsis leaves grown at aCO_2_ (**Figure 3A**); this may contribute to the lower wound-associated JA-Ile levels observed in WT plants grown at eCO_2_ or the lower JA-Ile levels in these plants may lead to lower ascorbate levels.

JA levels were higher in wounded *cyp20–3* plants grown at eCO_2_ compared to mutant plants grown at aCO_2_ or WT plants grown at either CO_2_ level (**Figure 1E**). At 90 min after wounding, JA-Ile levels in plants grown at eCO_2_ was notably lower than in plants grown at aCO_2_, this was particularly pronounced in WT plants (**Figure 1F**). This attenuation of the wound-induced jasmonate burst was reflected in lower wound- and jasmonate-responsive gene expression in plants grown at eCO_2_ compared with aCO_2_, a distinction abolished in the *cyp20–3* mutants (**Figure 4**).

Increased cysteine levels in stomatal guard cells may lead to the increased sulfation of molybdenum by the molybdenum cofactor sulfurase ABA3 (Watanabe et al., 2018). The resultant compound, molybdenum disulfide, is the cofactor for Arabidopsis aldehyde oxidase 3 (AAO3), which catalyses the conversion of abscisic aldehyde to ABA (Seo et al., 2000). Thus, stress-related increases in OPDA results in ABA biosynthesis in guard cells (Sun et al., 2025). Contrary to these findings, in wounded arabidopsis leaves, we see higher ABA levels in the *cyp20–3* mutant which likely reflects cell type-specific differences.

## Conclusions

In response to recognition of necrotrophic pathogens or chewing insect herbivores, a strong, robust increase in jasmonates leads to plant resistance (Koo and Howe, 2009; Li et al., 2022). However, this jasmonate burst is attenuated in some C3 plants grown at future predicted atmospheric eCO_2_ levels (Bazinet et al., 2022 and references therein). Given the importance of jasmonate signaling in plant resistance, understanding the underlying mechanism(s) is critical.

Even though foliar levels of JA and/or JA-Ile are lower in wounded arabidopsis grown at eCO_2_, levels of the precursor to these jasmonates, OPDA, are not affected by the atmospheric CO_2_ level. Thus, we focused on the role of the OPDA-binding protein CYP20–3 in regulating stress-associated jasmonate levels at eCO_2_. Our results suggest that CYP20–3 has a two-tier role in regulating wound-associated jasmonate biosynthesis in C3 plants grown at eCO_2_. First, we propose that the decreased photorespiration of C3 plants in the presence of eCO_2_ increases the availability of electrons from the pETC to be passed to CYP20-3, activating the formation of the CYP20-3-OPDA-SAT1-OASTL-B complex to limit OPDA flux into JA-Ile biosynthesis in wounded plants. Secondly, through the action of the CYP20-3-OPDA-SAT1-OASTL-B complex, cysteine biosynthesis is enhanced that leads to glutathione to buffer changes in the cellular redox state and limit the wound-associated activation of jasmonate biosynthesis. Although there is variability reported (Bazinet et al., 2022), decreased resistance to necrotrophic pathogens and caterpillar herbivory that correlate to lower induced JA and JA-Ile levels has been reported in diverse C3 plant species grown at eCO_2_, such as tomato, melon, rice and alfalfa (Guo et al., 2012; Lu et al., 2018; Hu et al., 2020; Johnson et al., 2020; Zhang et al, 2020), suggesting that the proposed CYP20-3-OPDA regulatory mechanism may be conserved in C3 crops.

## Data Availability

All data generated from this research are presented in the results or are available in the **Supplementary Material** of this article. Raw read data (FASTQ) have been deposited in the NCBI Sequence Read Archive (Bioproject ID PRJNA1332053).

## Supplementary material

Supplementary Figure 1 CYP20–3 transcript and protein levels. *Arabidopsis thaliana*, wildtype (WT) and *cyp20-3* (*cyp*) were grown at ambient (aCO_2_, 450 ppm) or elevated (eCO_2_, 900 ppm) carbon dioxide. Rosettes were either left undamaged or wounded when plants reached growth stage 3.9 (Boyes et al., 2001). **(A)** *AtCYP20–3* expression measured by RNA-Seq. **(B)** Western blot. Proteins were extracted from frozen rosettes and equal masses of protein were separated by SDS-PAGE. A molecular weight marker was included in the first well: Spectra Multicolor Broad Range Protein Ladder (Thermo Fisher). CYP20-3 (20 kDa) was detected by immunoblot with an anti-CYP20–3 antibody (PhytoAB).

Supplementary Figure 2 *Arabidopsis thaliana cyp20–3* plants are smaller than wildtype. Wildtype (WT) and *cyp20–3* plants were grown at ambient CO_2_ (aCO_2_, 450 ppm) and elevated CO_2_ (eCO_2_, 900 ppm). Rosettes were either left undamaged (U) or wounded (W) when plants reached growth stage 3.9 (Boyes et al., 2001). Biomass was analyzed by 2-factor analysis of variance (ANOVA) (Factors: CO_2_, genotype) followed by Tukey HSD *post-hoc* test to identify significant differences. **(A)** WT and *cyp20–3* mutants grown at aCO_2_ or eCO_2_. **(B)** *cyp20–3* were 38% smaller than WT (F_(1,68)_ = 45.03, p < 0.001; **Supplementary Table 3**); on average, 20% of rosette biomass was removed by wounding the largest 7 rosette leaves on individual plants with a hole punch ensuring that the midvein was not damaged (**Supplementary Table 4**). Bar graphs represent the mean ± the standard error. Number signs (#) represent differences between unwounded and wounded plants. Asterisks (*) represent genotype differences at each CO_2_ level.

Supplementary Figure 3 CO_2_ and genotype affect abscisic acid levels in *Arabidopsis thaliana* rosettes. Wildtype (WT) and *cyp20–3* plants were grown at ambient CO_2_ (aCO_2_, 450 ppm) and elevated CO_2_ (eCO_2_, 900 ppm). Rosettes were either left undamaged or wounded when plants reached growth stage 3.9 (Boyes et al., 2001). Abscisic acid (ABA) was measured by ultrahigh performance liquid chromatography-mass spectrometry. Phytohormone levels in undamaged or damaged plants were analyzed by 3-factor analysis-of-variance (ANOVA) (Factors: CO_2_, genotype, time) followed by Tukey HSD *post-hoc* tests to identify significant differences (**Supplementary Table 5**). ABA levels are **(A)** lower in undamaged WT plants grown at eCO_2_, and **(B)** higher in *cyp20–3* compared to WT in wounded plants. Data points represent the mean ± standard error. The temporal increase in ABA levels in response to wounding is indicated by alphabetical letters.
