# Supplementary figures and images for: Interaction with CYP20–3 limits OPDA flux into jasmonate biosynthesis in wounded leaves of arabidopsis grown at eCO2
Source: Front Plant Sci. 2026 Apr 7;17:1725249. doi: 10.3389/fpls.2026.1725249 (PMC13096701; doi:10.3389/fpls.2026.1725249)

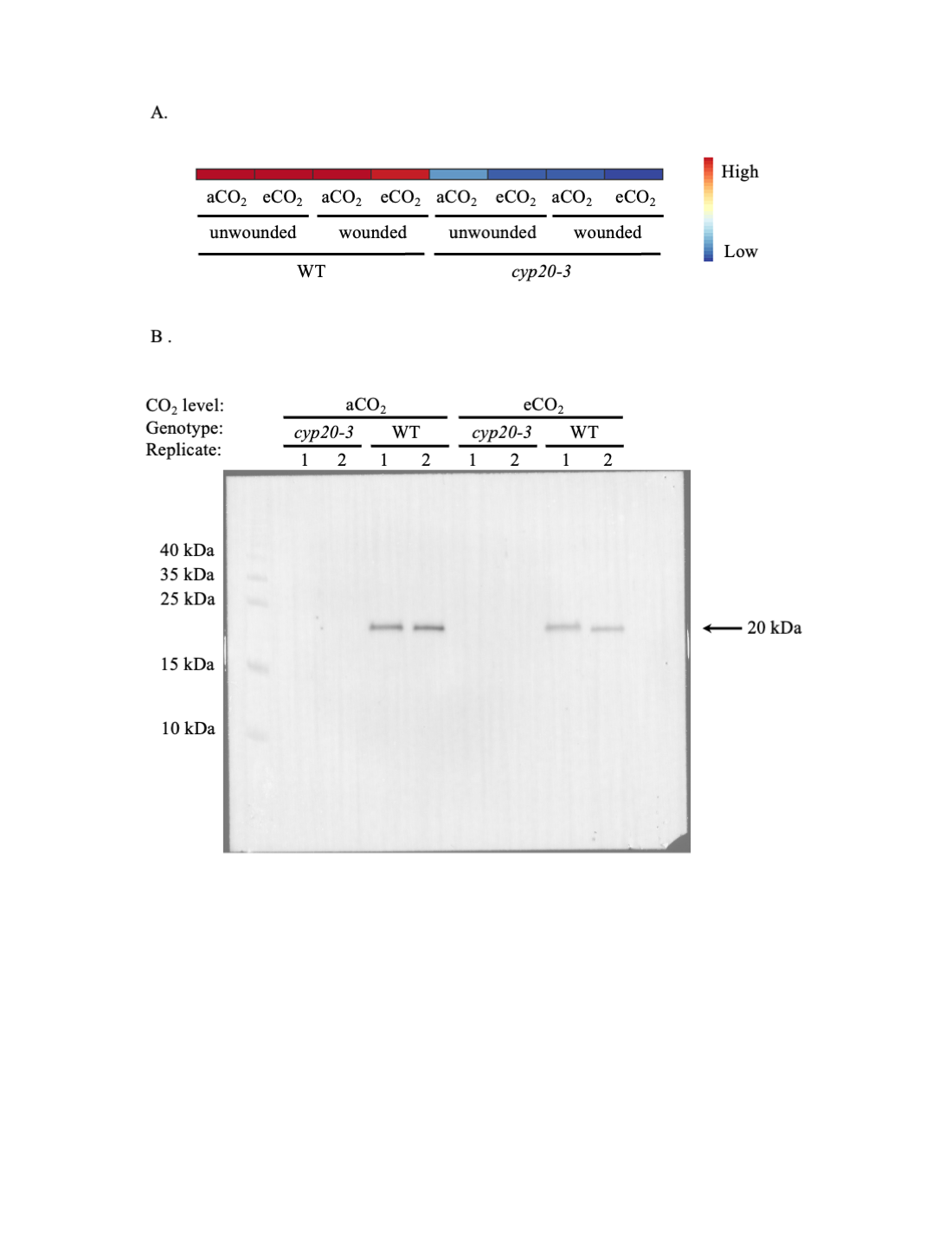

Supplement: Supplementary Figure 1 — CYP20–3 transcript and protein levels. Arabidopsis thaliana, wildtype (WT) and cyp20-3 (cyp) were grown at ambient (aCO2, 450 ppm) or elevated (eCO2, 900 ppm) carbon dioxide. Rosettes were either left undamaged or wounded when plants reached growth stage 3.9 (Boyes et al., 2001). (A) AtCYP20–3 expression measured by RNA-Seq. (B) Western blot. Proteins were extracted from frozen rosettes and equal masses of protein were separated by SDS-PAGE. A molecular weight marker was included in the first well: Spectra Multicolor Broad Range Protein Ladder (Thermo Fisher). CYP20-3 (20 kDa) was detected by immunoblot with an anti-CYP20–3 antibody (PhytoAB). [file Image1.tiff]

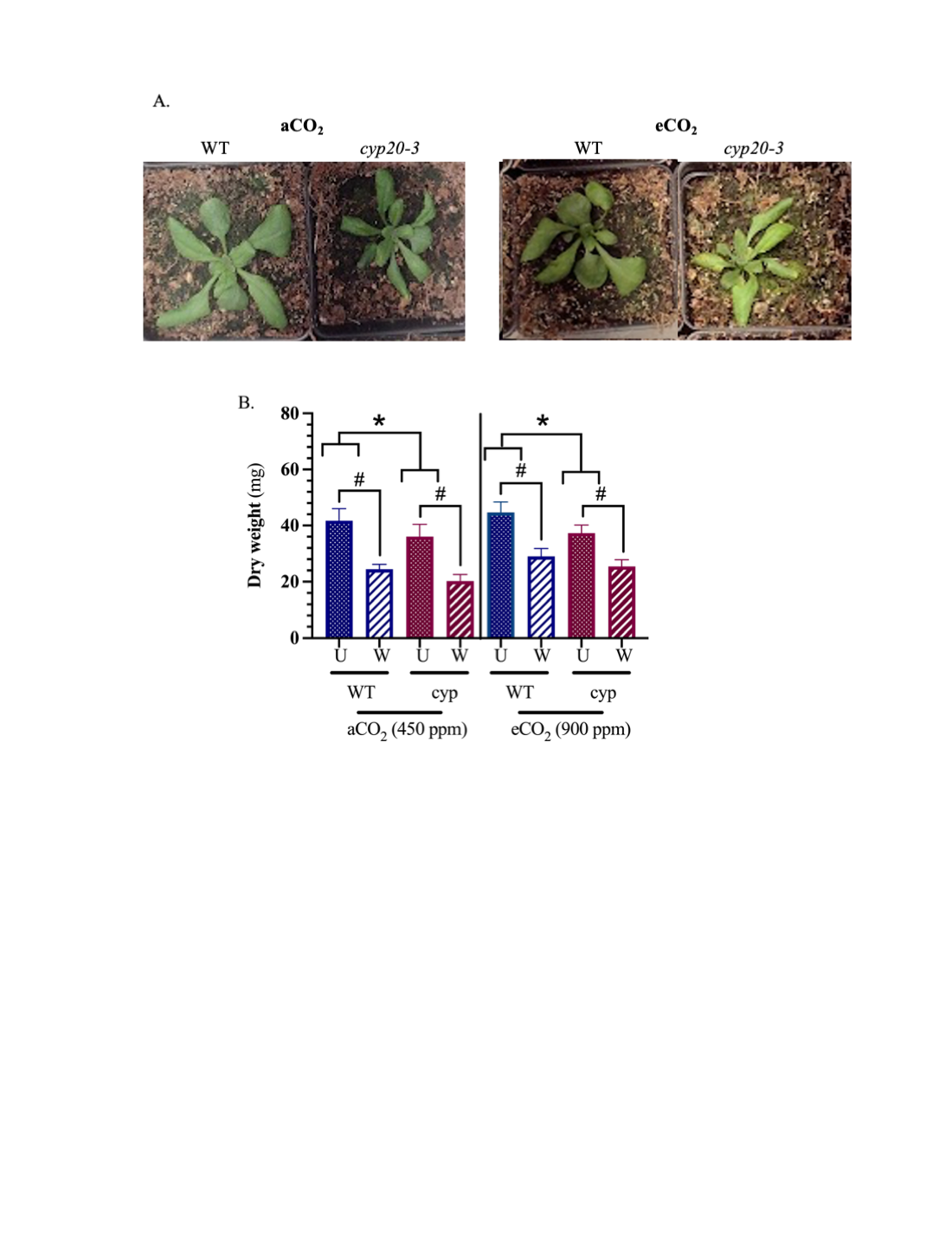

Supplement: Supplementary Figure 2 — Arabidopsis thaliana cyp20–3 plants are smaller than wildtype. Wildtype (WT) and cyp20–3 plants were grown at ambient CO2 (aCO2, 450 ppm) and elevated CO2 (eCO2, 900 ppm). Rosettes were either left undamaged (U) or wounded (W) when plants reached growth stage 3.9 (Boyes et al., 2001). Biomass was analyzed by 2-factor analysis of variance (ANOVA) (Factors: CO2, genotype) followed by Tukey HSD post-hoc test to identify significant differences. (A) WT and cyp20–3 mutants grown at aCO2 or eCO2. (B) cyp20–3 were 38% smaller than WT (F(1,68) = 45.03, p < 0.001; Supplementary Table 3); on average, 20% of rosette biomass was removed by wounding the largest 7 rosette leaves on individual plants with a hole punch ensuring that the midvein was not damaged (Supplementary Table 4). Bar graphs represent the mean ± the standard error. Number signs (#) represent differences between unwounded and wounded plants. Asterisks (*) represent genotype differences at each CO2 level. [file Image2.tiff]

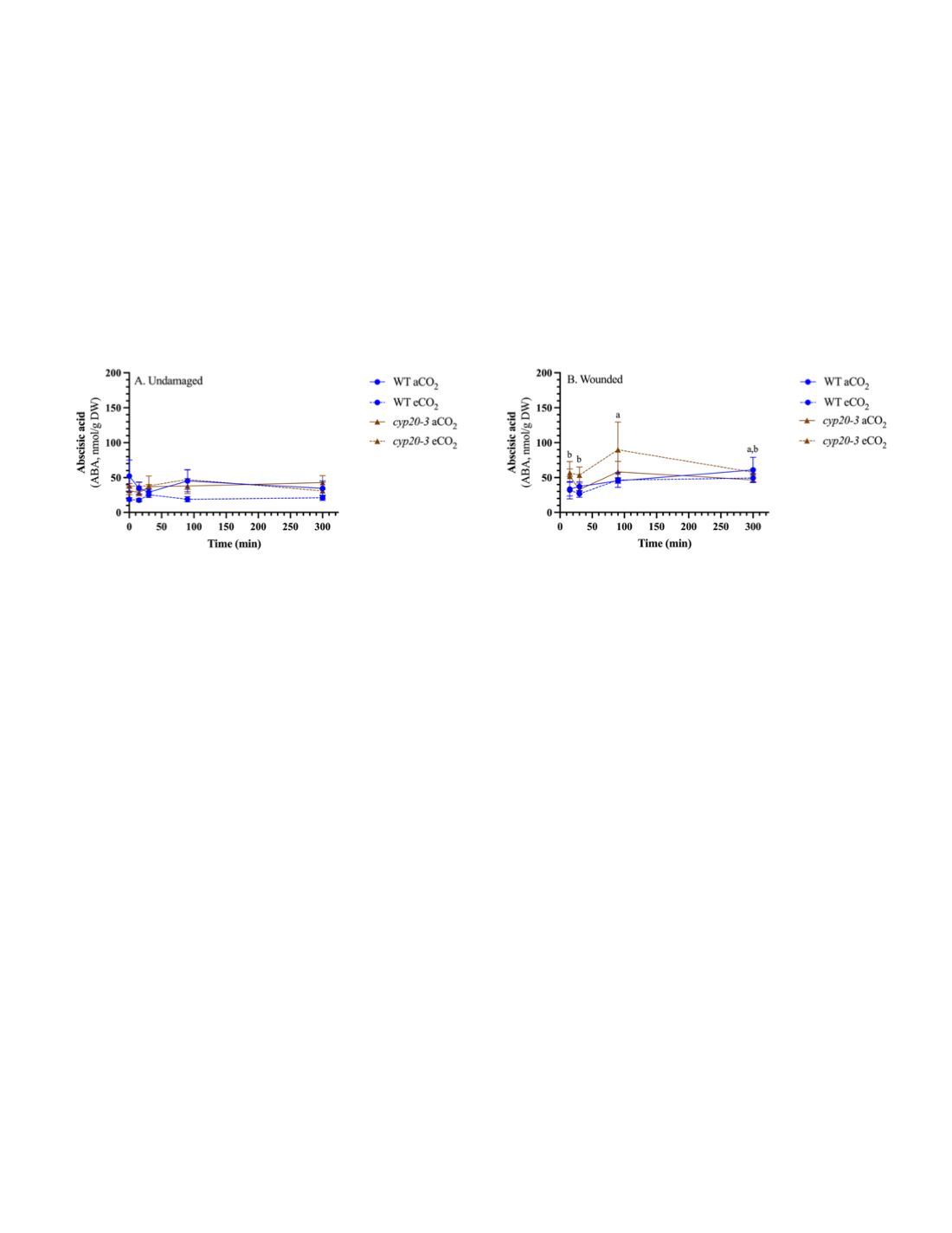

Supplement: Supplementary Figure 3 — CO2 and genotype affect abscisic acid levels in Arabidopsis thaliana rosettes. Wildtype (WT) and cyp20–3 plants were grown at ambient CO2 (aCO2, 450 ppm) and elevated CO2 (eCO2, 900 ppm). Rosettes were either left undamaged or wounded when plants reached growth stage 3.9 (Boyes et al., 2001). Abscisic acid (ABA) was measured by ultrahigh performance liquid chromatography-mass spectrometry. Phytohormone levels in undamaged or damaged plants were analyzed by 3-factor analysis-of-variance (ANOVA) (Factors: CO2, genotype, time) followed by Tukey HSD post-hoc tests to identify significant differences (Supplementary Table 5). ABA levels are (A) lower in undamaged WT plants grown at eCO2, and (B) higher in cyp20–3 compared to WT in wounded plants. Data points represent the mean ± standard error. The temporal increase in ABA levels in response to wounding is indicated by alphabetical letters. [file Image3.tiff]
